# UM171 suppresses breast cancer progression by inducing KLF2

**DOI:** 10.1007/s10549-024-07372-0

**Published:** 2024-06-14

**Authors:** Xiaojuan Ran, Anling Hu, Yi Kuang, Chunlin Wang, Wuling Liu, Xiao Xiao, Eldad Zacksenhaus, Xiangdi Yu, Yaacov Ben-David

**Affiliations:** 1 Guizhou University of Traditional Chinese Medicine, Guiyang, China; 2 Anesthesiology Department of Liuzhou People’s Hospital affiliated to Guangxi Medical University, Liuzhou, 545000 Guangxi China; 3 State Key Laboratory for Functions and Applications of Medicinal Plants, Guizhou Medical University, Guiyang, 550014 Guizhou People’s Republic of China; 4 The Natural Products Research Center of Guizhou Province, Guiyang, Guizhou People’s Republic of China; 5 Department of Medicine, Laboratory Medicine & Pathobiology and Medical Biophysics, University of Toronto, Toronto, ON M5G 1L1, Canada; 6 Division of Advanced Diagnostics, Toronto General Research Institute, University Health Network, Toronto, ON Canada

**Keywords:** UM171, Breast cancer, Proliferation, PIM1-PIM3 activation, KLF2, P21

## Abstract

**Purpose:**

Breast cancer is the most frequent cancer in women with significant death rate. Morbidity is associated with drug resistance and metastasis. Development of novel drugs is unmet need. The aim of this study is to show potent anti-neoplastic activity of the UM171 compound on breast cancer cells and its mechanism of action.

**Methods:**

The inhibitory effect of UM171 on several breast cancer (BC) cell lines was examined using MTT and colony-forming assays. Cell cycle and apoptosis assays were utilized to determine the effect of UM171 on BC cell proliferation and survival. Wound healing scratch and transwell migration assays were used to examine the migration of BC cell lines in culture. Xenograft of mouse model with 4T1 cells was used to determine inhibitory effect of UM171 in vivo. Q-RT-PCR and western blotting were used to determine the expression level of genes effected by UM171. Lentivirus-mediated shRNAs were used to knockdown the expression of KLF2 in BC cells.

**Results:**

UM171 was previously identified as a potent agonist of human hematopoietic stem cell renewal and inhibitor of leukemia. In this study, UM171 was shown to inhibit the growth of multiple breast cancer cell lines in culture. UM171-mediated growth inhibition was associated with the induction of apoptosis, G2/M cell cycle arrest, lower colony-forming capacity, and reduced motility. In a xenotransplantation model of mouse triple-negative breast cancer 4T1 cells injected into syngeneic BALB/c mice, UM171 strongly inhibited tumor growth at a level comparable to control paclitaxel. UM171 increased the expression of the three *PIM* genes (PIM1-3) in breast cancer cells. Moreover, UM171 strongly induced the expression of the tumor suppressor gene *KLF2* and cell cycle inhibitor *P21*^*CIP*1^. Accordingly, knockdown of KLF2 using lentivirus-mediated shRNA significantly attenuated the growth suppressor activity of UM171. As *PIM1-3* act as oncogenes and are involved in breast cancer progression, induction of these kinases likely impedes the inhibitory effect of KLF2 induction by UM171. Accordingly, combination of UM171 with a PAN-PIM inhibitor LGH447 significantly reduced tumor growth in culture.

**Conclusion:**

These results suggested that UM171 inhibited breast cancer progression in part through activation of KLF2 and P21. Combination of UM171 with a PAN-PIM inhibitor offer a novel therapy for aggressive forms of breast cancer.

**Supplementary Information:**

The online version contains supplementary material available at 10.1007/s10549-024-07372-0.

## Introduction

With substantial advances in diagnosis and treatment, breast cancer (BC) is still a major cause of mortality in women. Despite conventional therapies, patients succumb to the disease due to metastasis, drug resistance, and tumor recurrence [1, 2]. Therefore, development of new drug targeting breast cancer cells is critical for eradication of this disease.

The pyrimido-indole derivative UM171 was previously developed to expand hematopoietic stem cells in mouse [3] and human [4]. UM171 induces expression of endothelial protein C receptor (EPCR/CD201/PROCR) in a small subset of human cord blood CD34 + cells that may be responsible for HSC expansion [5]. Another study demonstrated that knockdown of the Lysine-Specific histone demethylase 1A (LSD1/KDM1) in mice expands HSCs similar to the phenotype seen in UM171-treated mice [6], suggesting another possible target of UM171. UM171 was later shown to potentiate the activity of a CULLIN3-E3 ubiquitin ligase (CRL3) complex to degrade Kelch/BTB domain protein KBTBD4. UM171 activation of the CULLIN3-KBTBD4 ubiquitin ligase then specifically targets LSD1 corepressor complex for proteasomal degradation, inducing HSC expansion [7]. In a recent study, UM171 was shown by us to have a strong anti-leukemia activity [8]. In addition to LSD1, we identified proto-oncogene serine/threonine-protein kinase PIM1 as another target of UM171. Binding of UM171 to PIM1 activates this kinase resulting in HSC expansion [8]. Interestingly, anti-leukemic activity of UM171 is independent of LSD1 and PIM1 [8] and is likely mediated through another target. The anti-leukemic activity of UM171 is attributed to its upregulation of the tumor suppressor genes KLF2 and P21^*CIP*1^ [8].

Cancer stem cells (CSCs) represent a subpopulation of tumor cells with different responses to cancer therapy that may underlie relapse [9]. The HSC expansion induced by UM171 was shown to interfere with its independent anti-leukemia activity in a mouse model of leukemia [8]. Therefore, combining UM171 with a PAN-PIM inhibitor resulted in better inhibitory effect in leukemia [8]. To combat Leukemia stem cells (LSCs), many inhibitors of LSD1 were developed for treatment of this malignancy [10]. The combination of LSD1 inhibitors with other anti-cancer drugs could eliminate relapse and provide a better therapy [11]. Combining LSD1 with UM171 is then expected to significantly improve cancer therapy [8].

KLF2 is one of the 18 member of the Krüppel-like transcription factors containing highly conserved DNA-binding zinc finger domains. In addition to various biological functions, KLF2 has been shown to be involved in progression of various cancers [12–17]. In a previous study, UM171 compound was shown to strongly induce the expression of KLF2 in erythroleukemia cells and ablation of its expression significantly attenuated growth suppression by this compound. This growth inhibition by KLF2 in leukemic cells was shown to be mediated independence of its stem cell expansion activity [8]. These results were further supported by the tumor suppressor function of KLF2 in various cancers.

Herein, we investigated the effect of UM171 on breast cancer. We showed that UM171 exerts a strong anti-neoplastic activity on breast cancer cell lines in culture and in a xenotransplantation mouse model. UM171 triggered upregulation of the *PIM1-3* as well as KLF2, which in part mediated the anti-neoplastic activity of this compound. These results implicated UM171 as candidate drug for treatment of breast cancer and possibly other types of epithelial malignancies.

## Materials and methods

### Cells culture and compound treatment

The human and murine cell lines originated from breast cancer (MDA-MB-231, MCF-7 and 4T1), erythroleukemia (HEL), embryonic kidney (HEK293T) were all obtained from ATCC (US) and cultured as well as maintained in Dulbecco’s Modified Eagle Medium supplemented with 5% fetal bovine serum (HyClone, GE Healthcare, US).

For drug therapy, MDA-MB-231 cells (5 × 10^3^), 4T1 cells (5 × 10^3^), or MCF-7 (5 × 10^3^) cells were seeded on 96-well plates and then treated with the indicated concentrations of UM171 or DMSO as the vehicle control. After 0 h, 24 h, 48 h or 72 h, cells were incubated with MTT (5 mg/mL) reagent (Solarbio 298-93-1, CN) for 4 h. Next, cells were incubated with dissolved solution (10% SDS, 5% isobutanol, and 0.012 mol/L HCL) overnight. Finally, absorbance was subsequently measured at 570 nm using a spectrophotometer (Gene Company Limited, CN) and a proliferation index was constructed via MTT assay. The PAN-PIM inhibitor (LGH447) was purchased from APE-BIO (A89505, US).

### Cell cycle and apoptosis analyses

MDA-MB-231 cells (2 × 10^5^) or 4T1 cells (2 × 10^5^) were seeded on 6-well plates and then treated with the indicated concentrations of UM171 or DMSO as the vehicle control for 24 h. The morphology of cells was observed by inverted microscope (LEICA DMi8, Germany). For cell cycle analysis, obtained cells were fixed with 70% ethanol overnight at − 20 °C and then incubated with cycle staining buffer (PI: RNase A: TritonX-100 = 75:7.5:1) for 30 min at 37 °C. Cells were filtered using 200-mesh cell sieves and analyzed by flow cytometer (NovoCyte flow cytometer (ACEC Biosciences Inc., CA, US). For apoptosis analysis, cells were incubated with PI and Annexin V (BD FITC Annexin V Apoptosis Detection Kit I, #556547) for 15 min. Finally, cells were analyzed by flow cytometer using NovoCyte flow cytometer (ACEC Biosciences Inc., CA, US).

### Wound healing scratch assay

For wound healing scratch assay, MDA-MB-231 cells (2 × 10^5^) or 4T1 cells (2 × 10^5^) were seeded on 6-well plates. The next day, scratches were made by a sterile 200 μL pipette tip and cells were treated with indicated drugs at the same time. Pictures were taken using an inverted microscope at 0 h, 24 h, and 48 h. Then, the area of the scratches was analyzed and used to calculate the wound healing ratio (%).

### Transwell migration and clone-forming assay

For transwell migration, after treated with indicated drugs, cells (2 × 10^4^) were inoculated in the upper chamber, and complete medium was added in the lower chamber. After incubated for 24 h at 37 °C, cotton swabs were used to remove the cells at the upper surface of membrane. After fixing with 4% paraformaldehyde, cells were stained by crystal violet reagent (Beyotime Biotechnology, #C0121, CN) and observed by inverted microscope. For clone-forming assay, after treated with indicated drugs, cells (1 × 10^3^) were seeded on 6-well plates and cultured in complete medium for 14 days. After fixing with 4% paraformaldehyde, cells were stained by crystal violet reagent and observed by camera. Three discontinuous areas were used to analyze the counts of migration or clone forming.

### RNA extraction and Q-RT-PCR

Cells (2 × 10^6^) were seeded in 100 mm^2^ wells and treated with indicated drugs for 24 h. As described in previous study, we used the same procedures to extract RNA using TRIzol regent (Thermo Fisher Scientific, US) [8]. Then, RNA is reverse-transcribed into cDNA with the PrimeScript RT reagent kit (Takara Bio, CN) [8]. The Step One Plus Real-time PCR system (Thermo Fisher Scientific, US) was used for Q-RT-PCR as described previously [8], and the house-keeping gene *GAPDH* was set as a normalized control (100%). The primer sequences are listed in Table 1. All Q-RT-PCR experiments performed in three independent replicates for at least three biological replicates, each in triplicate (*n* = 3).

**Table 1:** Primers for real-time PCR

Gene	Forward sequence	Reverse sequence
H-GAPDH	GGAGCGAGATCCCTCCAAAAT	GGCTGTTGTCATACTTCTCATGG
H-KLF2	TTCGGTCTCTTCGACGACG	TGCGAACTCTTGGTGTAGGTC
H-P21	TGTCCGTCAGAACCCATGC	AAAGTCGAAGTTCCATCGCTC
H-PIM1	GGCTCGGTCTACTCAGGCA	GGAAATCCGGTCCTTCTCCAC
H-PIM2	TTGACCAAGCCTCTACAGGG	CCACCTGGAGTCGATCTGTGA
H-PIM3	AAGGACGAAAATCTGCTTGTGG	CGAAGTCGGTGTAGACCGTG

### Western blot analysis

Cells (1 × 10^6^) were seeded in 100 mm^2^ wells and treated with indicated drugs for 24 h. The proteins were then extracted using IP lysis for 30 min and used to detect by western blot. The bands were incubated with primary antibodies overnight at 4 °C and incubated with second antibodies for 2 h at room temperature. Finally, we used the Odyssey system (LI-COR Biosciences) to image and analyze the proteins, as described [18]. The antibodies are listed as follows: the GAPDH (AB-P-R001) antibody was obtained from Goodhere Biotech (CN); KLF2 antibody (222842) was obtained from ZEN-BIOSCIENCE (CN); goat anti-rabbit IgG (H + L) DyLight (TM) 800 (5151) and Anti-mouse IgG (H + L) DyLight (TM) 680 (5470) were obtained from Cell Signaling Technology (US).

### ShRNA lentiviral construction

ShKLF2 Vigene Bioscience (Shangdong, CN) and scrambled control plasmids were cloned into the unique BcuI sites of the PLent-GFP expression vector (Vigene Bioscience, US). Using Lipofectamine 2000 (11668–019, Thermo Fisher Scientific, US), shKLF2 (6 μg) was co-transfected with packaging plasmids psPAX2 (3 μg) and pMD2.G (6 μg) (Addgene plasmid #12259 and #12260) into HEK293T cells for producing shRNA lentiviruses. After 48-h post-transfection, MDA-MB-231 cells were transduced with the supernatants, which were harvested and filtered through 0.45 µm filters. After 48 h, puromycin-resistant cells were selected by adding 5 mg/mL puromycin (Solarbio, Beijing, CN) in the medium. The sequences of shRNA are listed in Table 2.

**Table 2:** KLF2 ShRNA Sequence

	KLF2 shRNA
shRNA1	CGGCACCGACGACGACCTCAATTCAAGAGATTGAGGTCGTCGTCGGTGCCGTTTTTT
shRNA2	AGTTCGCATCTGAAGGCGCATTTCAAGAGAATGCGCCTTCAGATGCGAACTTTTTTT
shRNA3	CACCGGCCATTCCAGTGCCATTTCAAGAGAATGGCACTGGAATGGCCGGTGTTTTTT

### 4T1 breast cancer model and drug therapy

BALB/c females (5–6 weeks old) were purchased from Tengxin (SCXK2019-0010) (CN). After acclimation with the environment for a week, each mouse was implanted with 1 × 10^6^ 4T1 cells (100 μL of cell suspension) on the breast pat. At one week after cell inoculation, mice were intraperitoneally injected with UM171 (10 mg/Kg body weight) every other day for 7 times and paclitaxel (8 mg/Kg body weight) (MCE, HY-B0015, US) as the positive control. After a week of treatment, tumor volume (mm^3^) and body weight (*g*) were recorded for 28 days, when the tumor become smaller than the permitted size. The tumors were collected after mice were anesthetized with pentobarbital.

### Statistical analysis

The statistical analysis was conducted using a two-tailed Student t test or a one-way ANOVA with Tukey’s post hoc test, using Graphpad Prism software. The threshold for significance was indicated within the figures as follows: * (*P* =  < 0.05), ** (*P* =  < 0.001), *** (*P* =  < 0.0001), and **** (*P* =  < 0.00001). The results were expressed as mean ± the standard deviation from at least 3 independent experiments.

## Results

### UM171 inhibited growth of breast cancer cell lines in culture

**Fig. 1:**
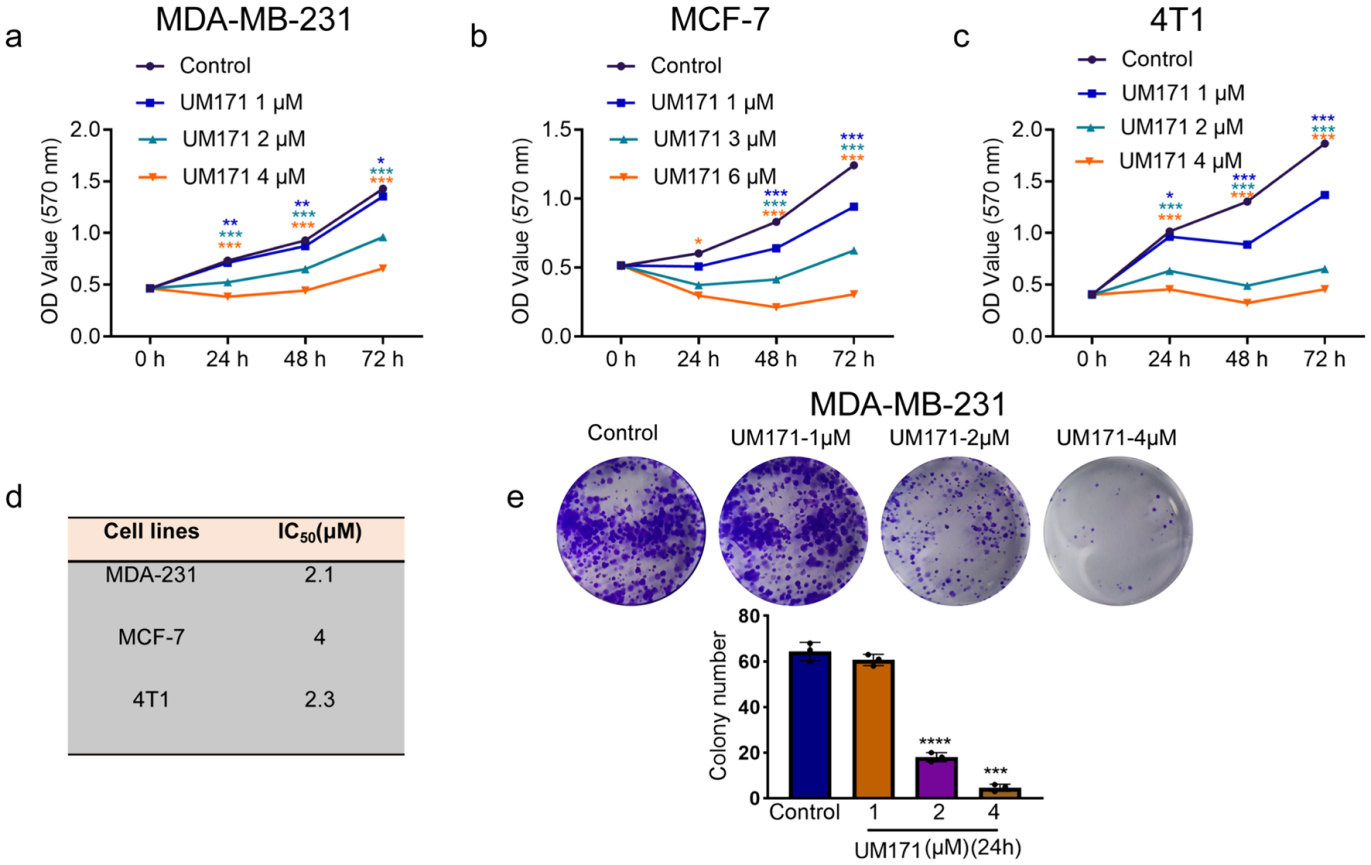
UM171 inhibited breast cancer cell proliferation in culture (**a**–**c**) Proliferation analysis of MDA-MB-231 (**a**), MCF-7 (**b**), and 4T1 (**c**) cells using the indicated doses of UM171, as determined by MTT assay. **d** The IC50 analysis of the indicated cell lines after treatment for 3 days with UM171. **e** MDA-MB-231 cells were treated with the indicated doses of UM171 for 24 h and plated for 14 days. Cells were then stained with crystal violet reagent and colonies were counted. Average of three independent experiments were shown below. *P* < 0.05 (*), *P* < 0.01 (**), *P* < 0.001 (***), and *P* < 0.0001 (****)

UM171 was previously shown to expand stemness and inhibit the growth of leukemic cells in culture and in vivo [8]. To study its effect on other types of cancer, we investigated the effect of UM171 on growth of several breast cancer cell lines. We found that UM171 effectively inhibited the proliferation of human MDA-MB-231 triple-negative (Fig. 1a) and MCF-7 luminal (Fig. 1b) as well as murine basal-like 4T1 (Fig. 1c) breast cancer cell lines in a dose-dependent manner. UM171 suppressed MDA-MB-231 proliferation with an IC50 of 2.1, while MCF-7 and 4T1 cells exhibited IC50 of 2.3 and 4, respectively (Fig. 1d). UM171 also strongly inhibited colony formation in a dose-dependent manner (Fig. 1e).

### UM171 altered cell cycle progression and promoted apoptosis of MDA-MB-231 and 4T1 cells in culture

**Fig. 2:**
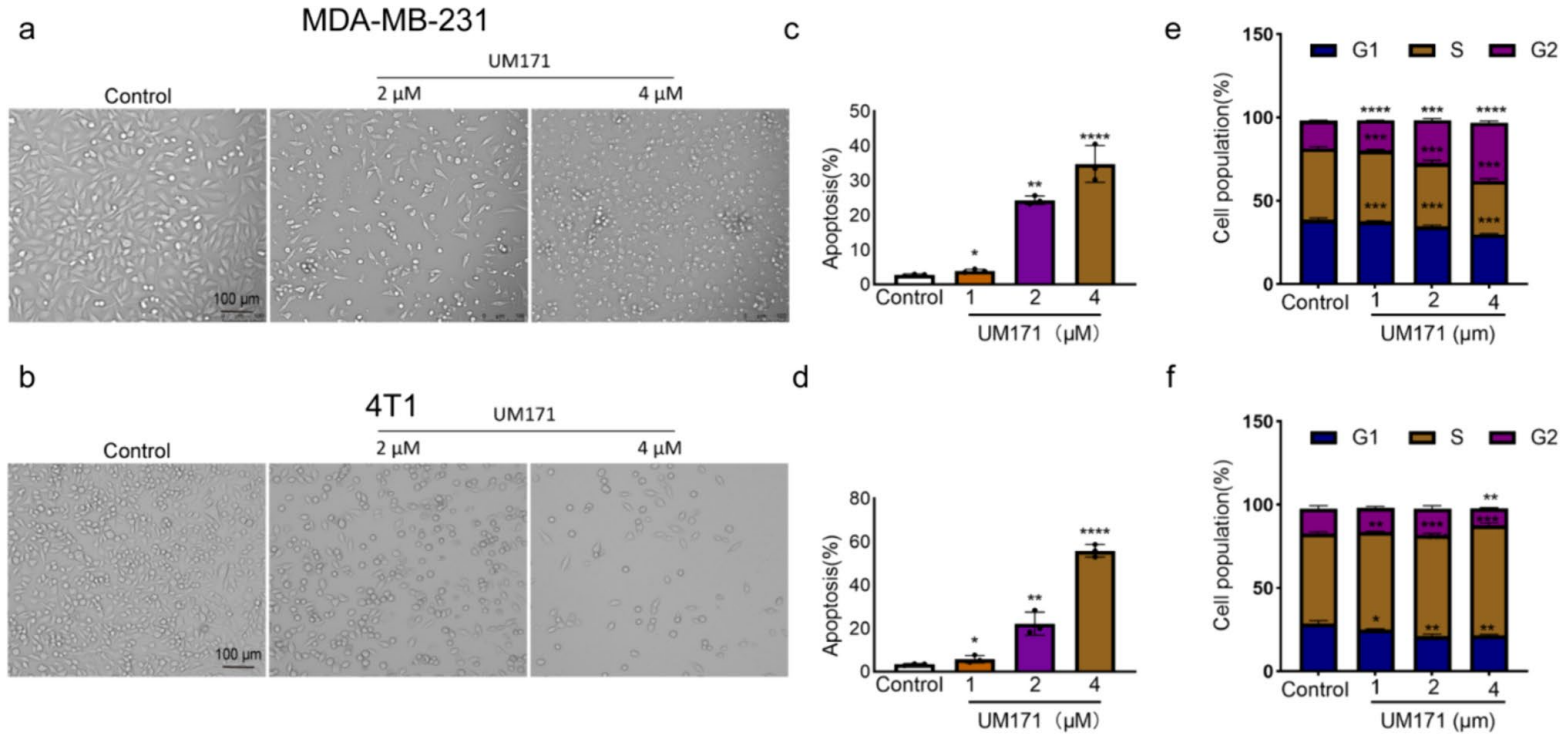
UM171 induced apoptosis and cell cycle arrest of breast cancer cells in culture (**a**, **b**) Microscopic images of MDA-MB-231 (**a**) and 4T1 (**b**) after treatment for 24 h with the indicated concentration of UM171 in culture. **c**, **d** The apoptosis index of MDA-MB-231 (**c**) and 4T1 (**d**) after treatment for 24 h with the indicated concentration of UM171. Average of three experiments were shown. **e**, **f** The cell cycle analysis of MDA-MB-231 (**e**) and 4T1 (**f**) cells after treatment with the indicated doses of UM171 for 24 h. Average of three experiments were shown. *P* < 0.05 (*), *P* < 0.01 (**), *P* < 0.001 (***), and *P* < 0.0001 (****)

UM171 induced dose-dependent cell death in both MDA-MB-231 and 4T1 cells, as appeared by microscopic examination (Fig. 2a, b) and Annexin V-PI flow cytometry apoptotic assays (Fig. 2c, d, supplemental Figs. 1a and 2a). Cell cycle analysis of MDA-MB-231 cells revealed that UM171 reduced the percentage of cells in G1, with concomitant increased in cells in the G2/M phase of the cell cycle (Fig. 2e and supplemental Fig. 1b). In contrast, UM171 caused no significant effect on cell cycle parameters of 4T1 cells (Fig. 2f and supplemental Fig. 2b).

### UM171 inhibited migration of breast cancer cells in culture

**Fig. 3:**
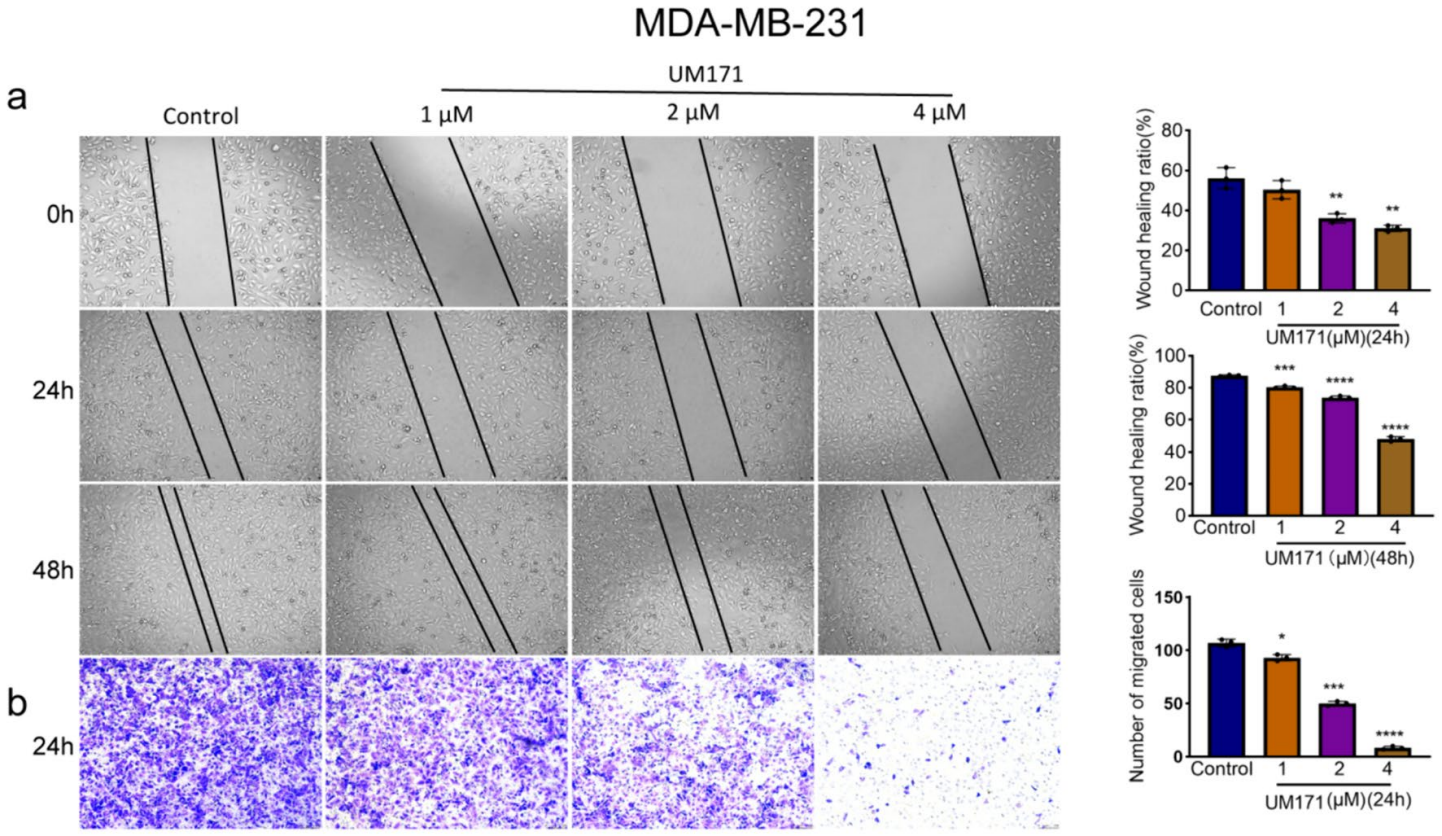
UM171 blocked migration of MDA-MB-231 cells (**a**) UM171 in a dose-dependent manner inhibited gap filling of the MDA-MB-231 cells for the indicated times in culture (Left panel). Average of three experiments for 24 h and 48 h is shown in the right panel. **b** Tumor migration assay of MDA-MB-231 cells over 24 h after staining with crystal violet reagent (Left panel). Average of three independent experiments was shown in the right panel. *P* < 0.05 (*), *P* < 0.01 (**), *P* < 0.001 (***), and *P* < 0.0001 (****)

**Fig. 4:**
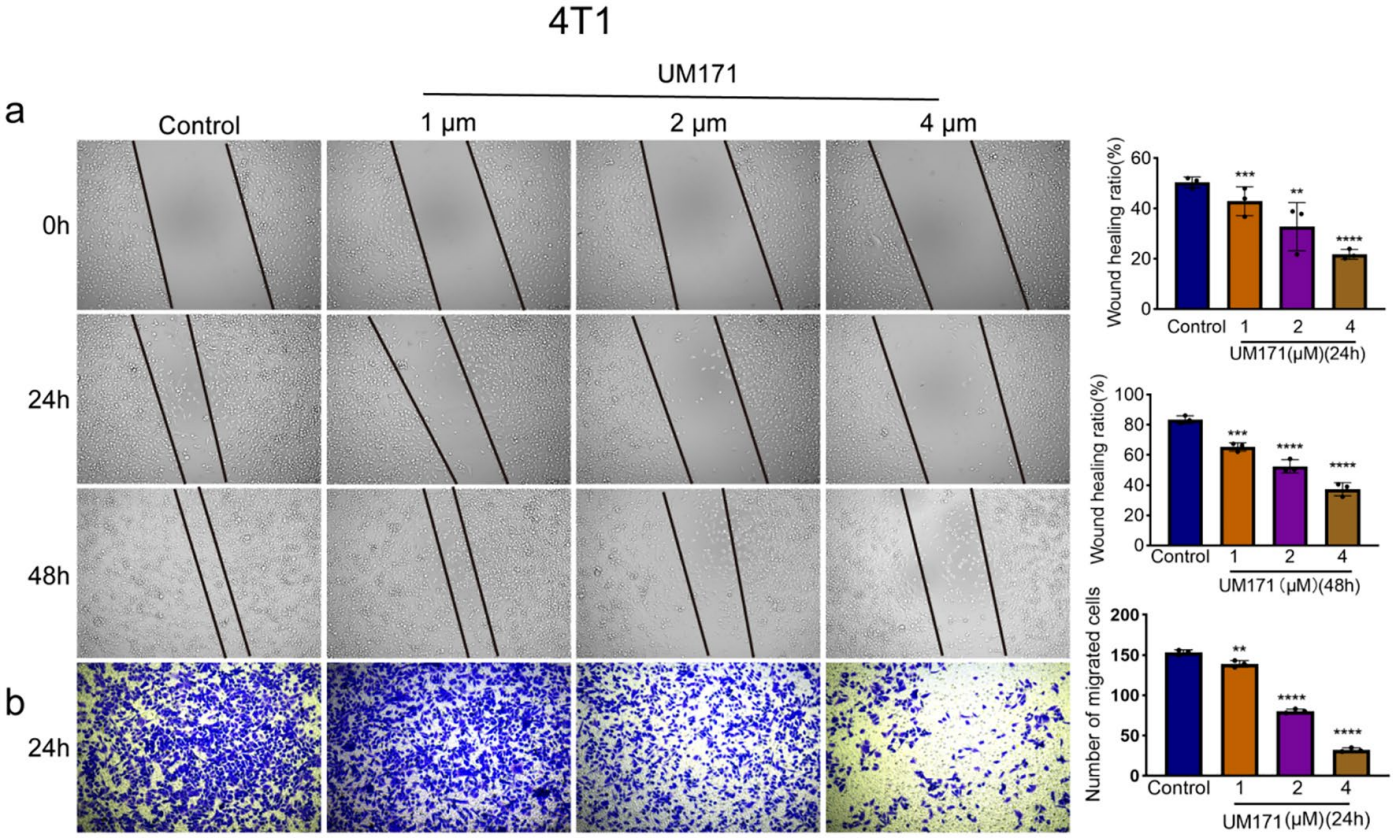
UM171 blocked migration of 4T1 cells (**a**) UM171 in a dose-dependent manner inhibited gap filling of the 4T1 cells for the indicated times in culture (Left panel). Average of three experiments for 24 h and 48 h is shown in the right panel. **b** Tumor migration assay of 4T1 cells over 24 h after staining with crystal violet reagent (Left panel). Average of three independent experiments is shown in the right panel. *P* < 0.01 (**), *P* < 0.001 (***), and *P* < 0.0001 (****)

To determine the effect of UM171 on cell migration, we next performed wound healing assay of MDA-MB-231 cells. UM171 significantly inhibited gap filling of the MDA-MB-231 cells in culture (Fig. 3a). Similar trend was also observed in tumor migration assay of MDA-MB-231 cells over 24 h in culture (Fig. 3b). A similar inhibition of tumor migration by UM171 was also observed in 4T1 cells (Fig. 4a, b). Together, these results revealed strong inhibition of both cell proliferation and migration by UM171 in breast cancer cells in culture.

### UM171 suppressed growth of 4T1 breast cancer cells in vivo

**Fig. 5:**
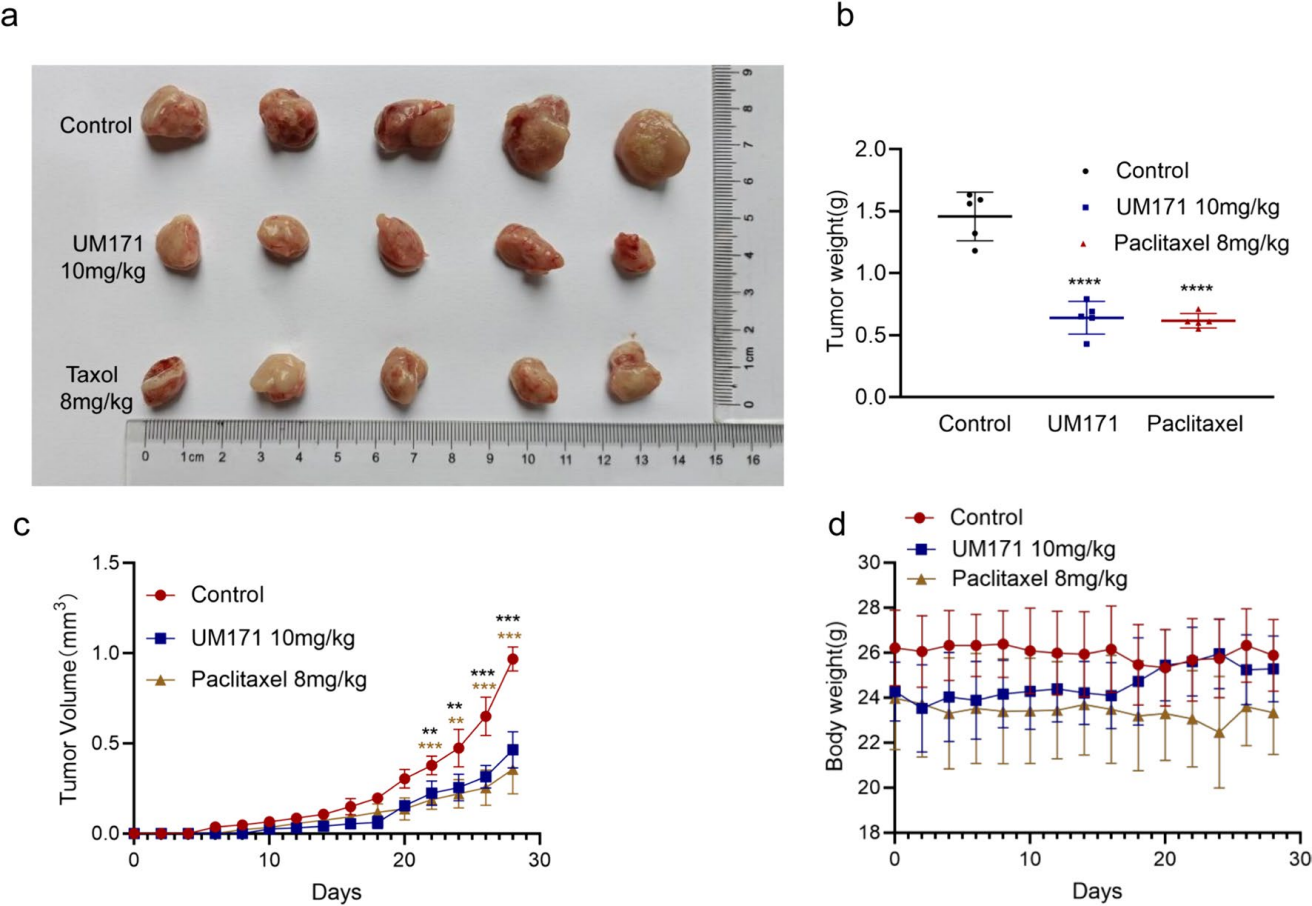
UM171 inhibited the progression of breast cancer 4T1 cells in vivo (**a**) BALB/c mice (*n*-5) were injected into the breast pads with 4T1 cells (1.0 × 10^6^). Seven days after 4T1 injection, mice were treated with UM171 (10 mg/kg) for 2 weeks (every other day). In this experiment, paclitaxel (8 mg/kg) was used as control. Tumors were removed at 28 day post-cell injection and photographed. **b** Tumor weight of control and treated groups. (C-D) The tumor volume (**c**) and body weight (**d**) of the control and injected mice at the indicated time period. *P* < 0.01 (**), *P* < 0.001 (***), and *P* < 0.0001 (****)

Due to strong inhibitory effect of UM171 of several breast cancer cell lines in culture (Figs. 1, 2, 3, 4), we examined the ability of this compound to attenuate the growth of murine 4T1 cells in xenotransplantation model in vivo. Groups of BALB/c mice were injected into the mammary fat pads with 4T1 cells (1 × 10^6^) and a week later were treated with UM171 (10 mg/kg) for 2 weeks (every other day). In this experiment, paclitaxel (8 mg/kg), commonly used for treatment of breast cancer [19], was used as positive control. Treatment with either UM171 or paclitaxel significantly inhibited growth of 4T1 in vivo (Fig. 5a–c). Whereas paclitaxel reduced body weight, albeit insignificantly, UM171 treatment did not significantly affect body weight, suggesting no overt side effects at this dose (Fig. 5d).

### UM171 induced the marker of breast cancer stemness and tumor suppressor genes KLF2 and P21^*CIP*1^

**Fig. 6:**
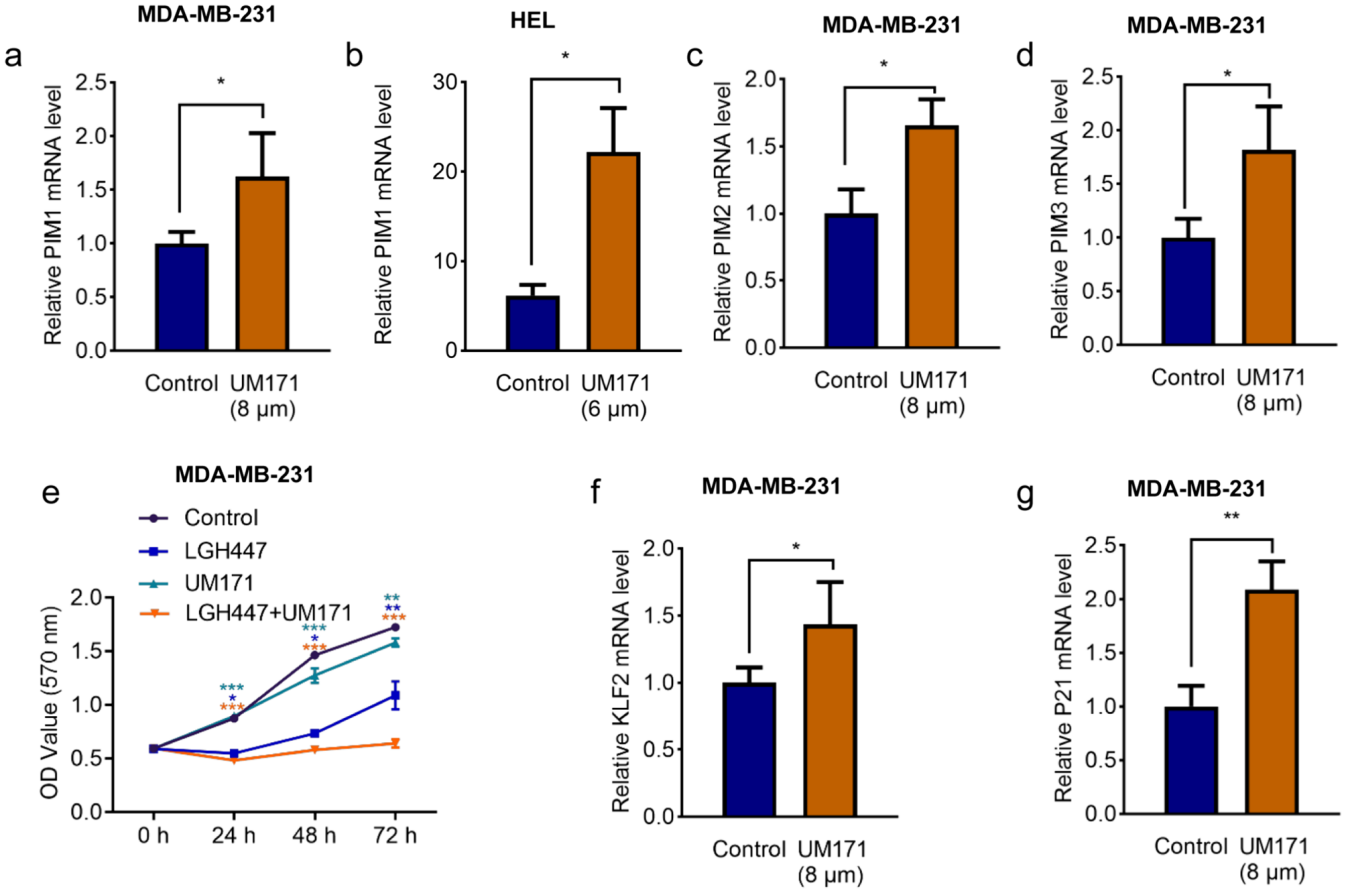
Induction of the PIM1-3 oncogenes and growth suppressor KLF2/P21 genes by UM171 in breast cancer cells (**a**–**d**) Expression of *PIM1* (**a**), *PIM2* (**c**), and *PIM3* (**d**) in MDA-MD-231 and *PIM1* in HEL (**b**) cells after treatment with UM171 for 24 h, as determined by Q-RT-PCR. **e** Growth rate of MDA-MB-231 cells treated with UM171 (2 μM), LGH447 (5 μM), and UM171 + LGH447, for the indicated time period. **f**, **g** The expression of *KLF2* (**f**) and *P21* (**g**) after treatment with UM171 for 24 h, as determined by Q-RT-PCR. *P* < 0.05 (*), *P* < 0.01 (**), *P* < 0.001 (***), and *P* < 0.0001 (****)

We previously showed that UM171 induced leukemia stem cell markers through binding to PIM1^8^. This binding caused activation and higher expression of PIM1 [8]. Higher expression of PIM1 was also seen in MDA-MB-231 cells treated with UM171 (Fig. 6a). However, much higher level of induction was seen in HEL erythroleukemic cells treated with UM171, as previously reported (Fig. 6b) [8]. Interestingly, UM171 also induced *PIM2* and *PIM3* expression, indicating activation of all three *PIM* genes by this agent (Fig. 6c, d). As PIM1-3 kinases are implicated in breast cancer progression [20–22], we examined the combined effect of UM171 and PAN-PIM inhibitor LGH447 on proliferation of MDA-MB-231 cells. While LGH447 alone significantly inhibited cell proliferation, combined treatment with UM171 + LGH447 further reduced cell growth in culture (Fig. 6e).

Induction of the tumor suppressor KLF2 and the cell cycle inhibitor P21^CIP1^ by UM171 independently of PIM1 has previously been shown to be responsible in part for its anti-leukemic activity [8]. Transcription of both *KLF2* and *P21* genes was induced in MDA-MB-231 cells treated with UM171 (Fig. 6f, g).

### UM171 inhibited cell proliferation in part through KLF2 induction

**Fig. 7:**
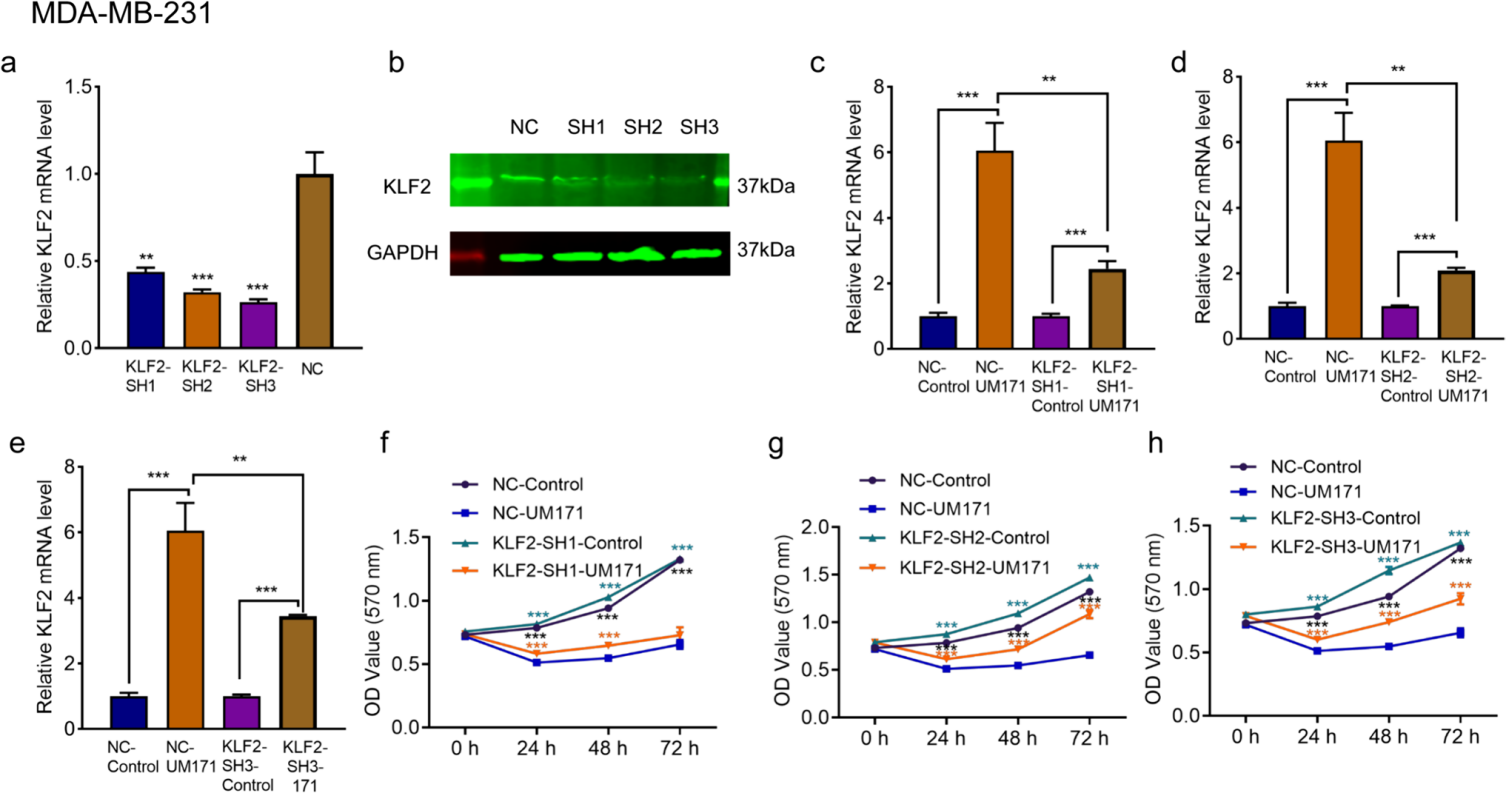
UM171 inhibited proliferation of breast cancer cells in part through the induction of *KLF2* (**a**, **b**). The expression of KLF2 in the lentivirus MD-MB-231 infected KLF2-sh1, KLF2-sh2, KLF2-sh3, and scrambled control cells, as determined by Q-RT-PCR (**a**) or western blotting (**b**). **c**–**e** The expression of KLF2 in KLF2-sh1 (**c**), KLF2-sh2 (**d**), and KLF2-sh3 (**e**) and their scrambled control group after treatment with DMSO or UM171 (2 μM), as determined by Q-RT-PCR. **f**, **h** The growth rate of KLF2-sh1 (**f**), KLF2-sh2 (**g**), KLF2-sh3 (**h**), and their corresponding control cells treated with or without UM171 (2 μM). *P* < 0.01 (**), *P* < 0.001 (***), and *P* < 0.0001 (****)

As *KLF2* expression was significantly induced in MDA-MB-231 cells (Fig. 6f), we examined whether growth inhibitory effect of UM171 was mediated through this tumor suppressor gene. To this end, we knocked down *KLF2* expression in MDA-MB-231 cells using lentivirus shRNA vectors. Expression of KLF2 was indeed significantly reduced in KLF2-sh1-, KLF2-sh2-, and KLF2-sh3-treated cells (Fig. 7a, b). KLF2 was robustly induced in control scrambled transfected cells, and was much reduced in KLF2-sh1 (Fig. 7c), KLF2-sh2 (Fig. 7d) and KLF2-sh3 (Fig. 7e) knockdown cells. Knockdown of KLF2 in MDA-MB-231 cells slightly, but significantly increased cell proliferation in KLF2-sh1 (Fig. 7f), KLF2-sh2 (Fig. 7g) and KLF2-sh3 (Fig. 7h) cells compared with control (NC-control) cells. When KLF2 knockdown cells were treated with UM171, the inhibitory rate of cell proliferation was significantly attenuated in KLF2-sh1 (Fig. 7f), KLF2-sh2 (Fig. 7g), and KLF2-sh3 (Fig. 7h) cells. These results suggested that activation of *KLF2* by UM171 mediated in part its growth inhibition.

## Discussion

The compound UM171 was previously shown to inhibit growth of leukemia cells in culture and in vivo through alteration in expression of specific genes [8]. In this study, we showed that UM171 could also inhibit the growth and migration of breast cancer cell lines in culture as well as the progression of mouse 4T1 breast cancer xenografts in vivo. The in vivo suppressor activity of UM171 was similar to the effect seen by paclitaxel, a potent drug commonly used for treatment of breast cancer [19]. UM171 blocked the proliferation of triple-negative breast cancer cell lines MDA-MB-231 and 4T1, suggesting an important therapeutic value for this pyrimido-indole derivative compound for treatment of this disease and likely other types of cancer.

Similar to leukemic cells, UM171 inhibited proliferation, induced apoptosis, and cell cycle arrest in breast cancer cells. While UM171 induced G2/M cell cycle arrest in MDA-MB-231 cells, no effect on cell cycle was seen when 4T1 cells were treated with this compound, suggesting different effects on cell cycle progression. In addition, UM171 strongly inhibited colony-forming ability and migration potential of breast cancer cells. These functions likely underline the strong suppression of 4T1 tumors in mice by UM171 and further implicate this compound as a candidate for the treatment of aggressive forms of breast cancer.

UM171 was previously identified as an agent which at low doses (nM) increases expansion of hematopoietic stem cells [3]. In breast cancer cell lines, UM171 was shown to increase the expression of the oncogenes PIM1-3, which are involved in the progression of breast cancer [20–22]. Thus, in addition to breast cancer inhibitory activity, UM171 also induces an oncogenic effect that should imped its anti-neoplastic activity. In the past decade, the *PIM* (1–3) genes were identified as targets for breast and other cancers, and accordingly, many PAN-PIM inhibitors were developed to combine with other cancer therapies [23–26]. Indeed, combining UM171 with PAN-PIM inhibitor LGH447 further inhibited cancer cell growth. Thus, combining UM171 with a PAN-PIM kinase inhibitors should provide an excellent therapy for breast and likely other cancer types.

In breast cancer cell lines, UM171 was shown to activate the expression of tumor suppressor gene KLF2 [13, 27] and cell cycle inhibitor P21 [28]. Indeed, KLF2 was previously implicated as a tumor suppressor gene in breast cancer [29]. Accordingly, lentivirus-mediated knockdown of *KLF2* in breast cancer cells significantly reduced growth suppression by UM171. These results implicated KLF2 as a potential target of UM171 that in part mediated growth suppression by this compound. As in leukemia, we have shown KLF2 induction independent of the PIM kinase pathway [8]. Further studies are needed to uncover the mechanism of induction of this tumor suppressor gene.

In conclusion, we have shown that UM171 exhibits a strong inhibitory activity in breast cancer cell lines associated with cell cycle arrest, apoptosis, lower migration, and colony-forming activities. This inhibitory activity of UM171 was also shown in xenografts of 4T1 breast cancer mouse model. While breast cancer inhibitory effect was in part associated with upregulation of *P21*^*CIP1*^ and *KLF2*, this compound also activated the expression of oncogenes PIM1-PIM3 that likely imped the anti-neoplastic of the compound. Therefore, combining UM171 with a PAN-PIM inhibitor could be used as a powerful tool for the treatment of triple-negative breast cancer and likely other type of malignancies.

### Supplementary Information

Below is the link to the electronic supplementary material.

Supplementary file1 (TIF 1471 KB)—Fig.1 UM171 induces apoptosis and cell cycle arrest of MDA-MB-231 breast cancer cells in culture (a) The apoptosis index of MDA-MB-231 cells after treatment for 24 h with the indicated concentration of UM171. (b) The cell cycle analysis of MDA-MB-231 cells after treatment with the indicated doses of UM171 for 24h.

Supplementary file2 (TIF 1018 KB)—Fig.2 UM171 induces apoptosis and cell cycle arrest of 4T1 breast cancer cells in culture (a) The apoptosis index of 4T1 cells after treatment for 24 h with the indicated concentration of UM171. (b) The cell cycle analysis of 4T1 cells after treatment with the indicated doses of UM171 for 24 h.

## Data Availability

Data available upon request.

## Abbreviations

BC: Breast cancer
EPCR/CD201/PROCR: Endothelial protein C receptor
LSD1/KDM1: Lysine-specific histone demethylase 1A
CRL3: CULLIN3-E3 ubiquitin ligase
CSCs: Cancer stem cells
LSCs: Leukemia stem cells

## Declarations

### Research involving human and animal participant

Animal care and procedures followed by the criteria for the use of laboratory animals. The animal protocol was reviewed and approved by the Guizhou Medical University Animal Care Committee under the guidelines of the China Council of Animal Care (Approval ID #1900373).
