# Supplementary figures and images for: UM171 suppresses breast cancer progression by inducing KLF2
Source: Breast Cancer Res Treat. 2024 Jun 14;207(2):405–15. doi: 10.1007/s10549-024-07372-0 (PMC11297059; doi:10.1007/s10549-024-07372-0)

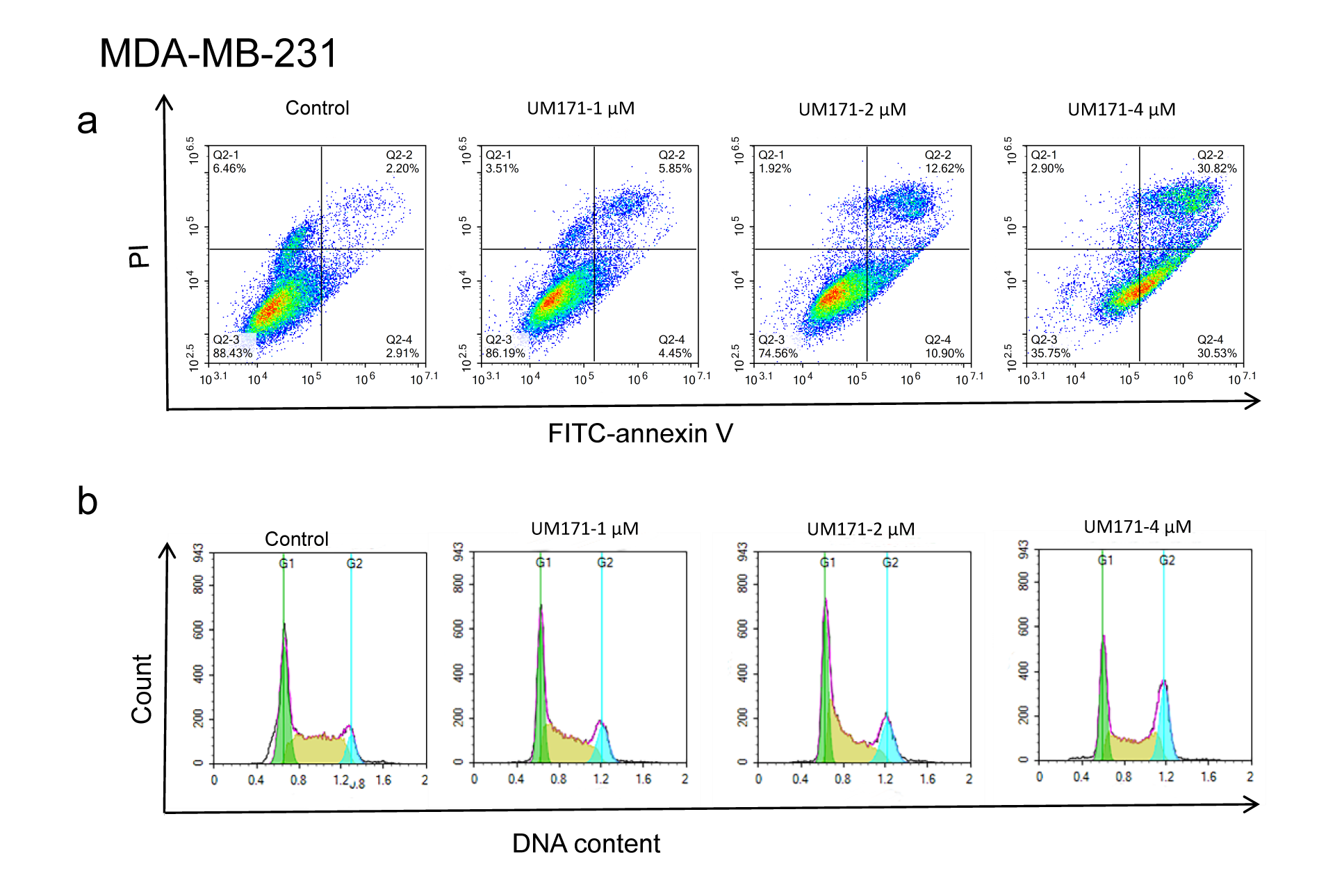

Supplement: Supplementary file 1 — Supplementary file1 (TIF 1471 KB)—Fig.1 UM171 induces apoptosis and cell cycle arrest of MDA-MB-231 breast cancer cells in culture (a) The apoptosis index of MDA-MB-231 cells after treatment for 24 h with the indicated concentration of UM171. (b) The cell cycle analysis of MDA-MB-231 cells after treatment with the indicated doses of UM171 for 24h. [file 10549_2024_7372_MOESM1_ESM.tif]

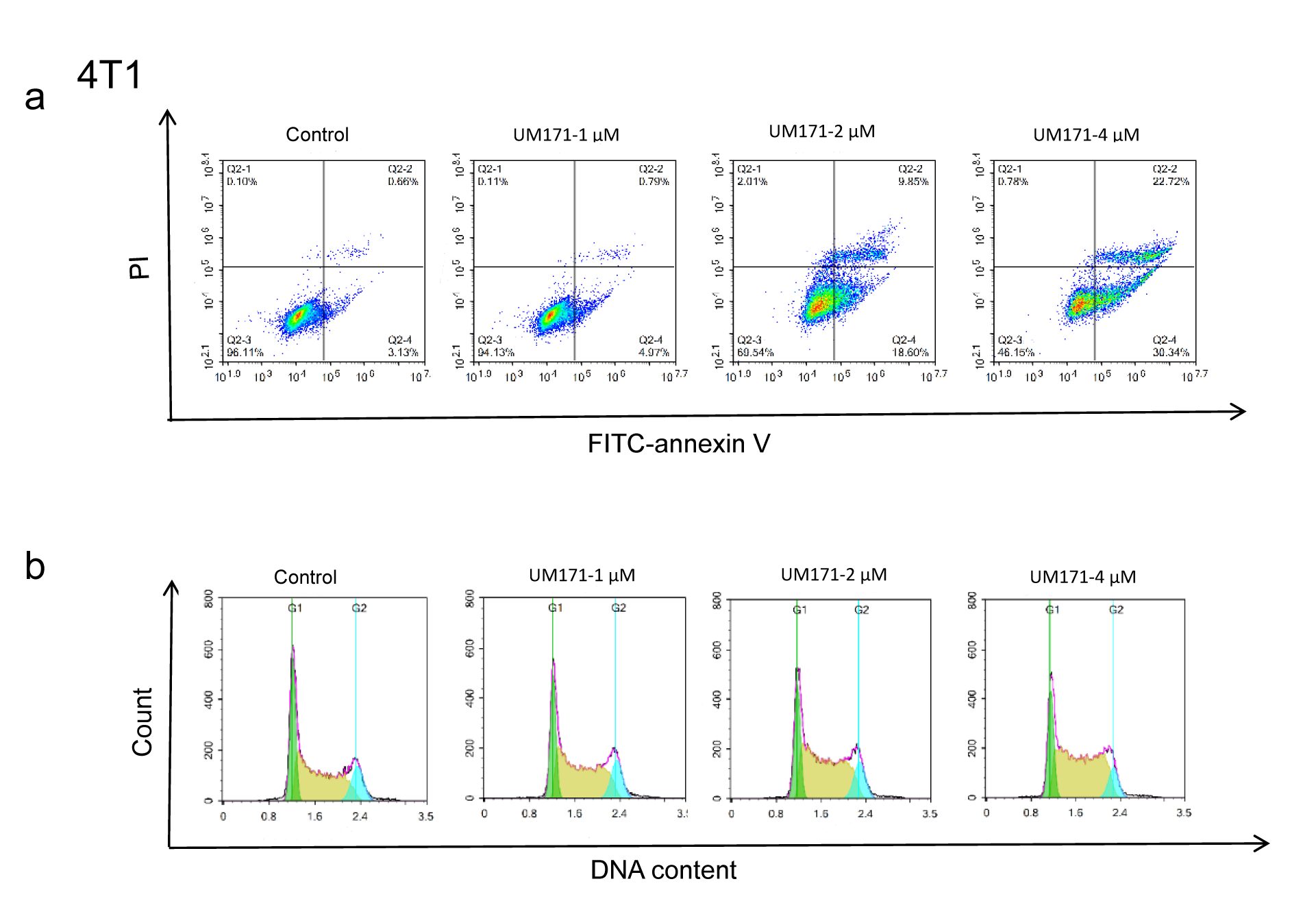

Supplement: Supplementary file 2 — Supplementary file2 (TIF 1018 KB)—Fig.2 UM171 induces apoptosis and cell cycle arrest of 4T1 breast cancer cells in culture (a) The apoptosis index of 4T1 cells after treatment for 24 h with the indicated concentration of UM171. (b) The cell cycle analysis of 4T1 cells after treatment with the indicated doses of UM171 for 24 h. [file 10549_2024_7372_MOESM2_ESM.tif]
